# iTRAQ-Based Quantitative Proteomics Analysis of Black Rice Grain Development Reveals Metabolic Pathways Associated with Anthocyanin Biosynthesis

**DOI:** 10.1371/journal.pone.0159238

**Published:** 2016-07-14

**Authors:** Linghua Chen, Yining Huang, Ming Xu, Zuxin Cheng, Dasheng Zhang, Jingui Zheng

**Affiliations:** 1 FujianAgriculture and Forestry University, Fuzhou Fujian, China; 2 Jinshan College of Fujian Agriculture and Forestry University, Fuzhou Fujian, China; 3 Department of Food and Biology Engineering, Zhangzhou Institute of Technology, Zhangzhou Fujian, China; 4 College of Crop Science, Fujian Agriculture and Forestry University, Fuzhou Fujian, China; 5 Shanghai Chenshan Plant Science Research Center, Shanghai Chenshan Botanical Garden, Chinese Academy of Sciences, Shanghai, China; 6 Shanghai Key Laboratory for Plant Functional Genomics and Resources, Shanghai, China; Institute of Botany, Chinese Academy of Sciences, CHINA

## Abstract

**Background:**

Black rice (*Oryza sativa* L.), whose pericarp is rich in anthocyanins (ACNs), is considered as a healthier alternative to white rice. Molecular species of ACNs in black rice have been well documented in previous studies; however, information about the metabolic mechanisms underlying ACN biosynthesis during black rice grain development is unclear.

**Results:**

The aim of the present study was to determine changes in the metabolic pathways that are involved in the dynamic grain proteome during the development of black rice *indica* cultivar, (*Oryza sativa* L. *indica* var. SSP). Isobaric tags for relative and absolute quantification (iTRAQ) MS/MS were employed to identify statistically significant alterations in the grain proteome. Approximately 928 proteins were detected, of which 230 were differentially expressed throughout 5 successive developmental stages, starting from 3 to 20 days after flowering (DAF). The greatest number of differentially expressed proteins was observed on 7 and 10 DAF, including 76 proteins that were upregulated and 39 that were downregulated. The biological process analysis of gene ontology revealed that the 230 differentially expressed proteins could be sorted into 14 functional groups. Proteins in the largest group were related to metabolic process, which could be integrated into multiple biochemical pathways. Specifically, proteins with a role in ACN biosynthesis, sugar synthesis, and the regulation of gene expression were upregulated, particularly from the onset of black rice grain development and during development. In contrast, the expression of proteins related to signal transduction, redox homeostasis, photosynthesis and N-metabolism decreased during grain maturation. Finally, 8 representative genes encoding different metabolic proteins were verified via quantitative real-time polymerase chain reaction (qRT-PCR) analysis, these genes had differed in transcriptional and translational expression during grain development.

**Conclusions:**

Expression analyses of metabolism-related protein groups belonging to different functional categories and subcategories indicated that significantly upregulated proteins were related to flavonoid and starch synthesis. On the other hand, the downregulated proteins were determined to be related to nitrogen metabolism, as well as other functional categories and subcategories, including photosynthesis, redox homeostasis, tocopherol biosynthetic, and signal transduction. The results provide valuable new insights into the characterization and understanding of ACN pigment production in black rice.

## Introduction

Rice (*Oryza sativa* L.) is one of the primary cereals of the world, serving as a major staple food source to more than half of the global population [1]. The most commonly consumed rice type is white rice; however, several special cultivars of rice consist of colored pigments such as red and black. Black rice presents a dark purple color, which is due to its high anthocyanin (ACN) content that is mainly secreted in the pericarp [2–4]. Black rice has been historically considered as a highly nutritious food in China and other East Asian countries [5]. Today, black rice is becoming increasingly popular in the US, Australia, as well as Europe, particularly in terms of its health benefits.

ACNs pertain to water-soluble flavonoids and are the primary pigments of black and red grains, as well as various other fruits, vegetables, cereals, and flowers [6]. In plants, ACNs serve as pollinators and seed dispersers, as well as photoprotectants that scavenge free radicals that are generated during photosynthesis [7]. Recent interest in ACNs as health-promoting food ingredients has mainly been due to its reported antioxidant activity [8, 9], which may contribute to the prevention of chronic and degenerative diseases [10–13]. Previous reports have also shown that ACNs also possess anti-inflammatory properties prevent arteriosclerosis [14] and hyperlipidemia [15, 16], lower glycemic indices [17], promote visual acuity[18], and hinder obesity and diabetes [19]. In addition, animal studies showed that an ACN-rich extract derived from black rice showing a relatively high ACN content (43.2%) imparted similar effects [20].

The involvement of ACNs in various important functions poses questions on how these compounds are produced, as well as regulated. In the past 3 decades, intensive studies have improved our understanding of ACNs in plant biology, including the biosynthesis and regulation of ACNs with other metabolites in various plant species such as *Arabidopsis*[20], maize[21], grape[22], orange[23], and apple[24], and majority of these ACN biosynthesis genes of interest has been cloned[25–28]. However, the synthesis of ACNs in black rice remains poorly understood and rarely studied despite its classification as a model crop plant and its genome being completely sequenced[29]. Furthermore, certain cultivars that contain high levels of ACNs have been developed, and these efforts facilitate in better understanding the activities relating to this particular biosynthetic pathway in black rice. Cyanidin-3-glucoside (C3G) and peonidin-3-glucoside (P3G), which are the predominant pigments in black rice, are the most powerful among the 14 main ACNs [30–33]. The cultivar (cv.) Heugjinju has the highest ACN and C3G levels among the dark purple rice cultivars [3, 29, 34].

Proteomics studies are generally conducted to obtain a comprehensive evaluation of proteins that respond to a specific environment or treatment, and these investigations provide both quantitative and active information, and protein-protein interactions[35]. Proteomic analysis of rice grains was previously reported, and the protein spots were identified by mass spectrometry as 309[36], 298[37], and 54[38], respectively. However, these investigations were performed using gel-based techniques, which limits the detection of proteins within grain proteomes. One disadvantage of this gel-based procedure is that the markedly over-represented group of grain-specific proteins may mask low-level proteins, thereby rendering its detection difficult [39]. Moreover, membrane proteins with several membrane spanning domains are difficult to detect on classical two-dimensional electrophoresis (2-DE) gels due to its low abundance as well as poor solubility in aqueous media[40]. The recently developed isobaric tags for relative and absolute quantitation (iTRAQ) proteomic method allow the unbiased and simultaneous observation of relative protein abundances within a cell during harvest [41–43]. Therefore, the iTRAQ method is suitable for investigating proteomic changes under various developmental processes[44]. iTRAQ was recently utilized in plant fruit development research of wheat [45]and grape[46–48]. Interestingly, iTRAQ has been determined to be a potentially useful approach in rice grain development research, as indicated by the results of a recent study on the mechanistic responses of inferior spikelets to drought stress [49].

In the present study, iTRAQ was utilized in the proteomic analysis of black rice grain to reveal the unique genetic profile, as well as identify alterations in the levels of protein expression during development. The goal of this study was to reveal differentially expressed proteins at five time points during the development of black rice grains (*O*. *sativa* L. *indica* var. SSP), as well as compare these protein expression patterns to identify candidate proteins that may be potentially involved in ACN biosynthesis. Furthermore, specific proteins were selected to correlate whether alterations in protein expression may be validated using transcript analysis. The results of the present study provide global insights into proteome modifications in black rice during development.

## Materials and Methods

### Plant materials and sampling

Black rice (*O*. *sativa* L. *indica* var. SSP) plants were cultured during the rice growing season (May to September) under natural and normal field conditions at the Fujian Agriculture University experimental field and were fertilized (urea, 60 kg/ha) using routine procedures. In rice, developing seeds are categorized as superior or inferior based on its location on spikes[50]. The superior seeds, which are located at the topmost region of the spike, generally show a higher growth rate, as well as simultaneously reach maturity. Therefore, in the present study, superior seeds were selected as experimental samples based on its distinguishing advantage of synchronous development [36]. The superior seeds of the topmost 3 spikelets were then labeled at noon during anthesis, when at least 50% of the superior seeds belonging to the corresponding spikelets were at the flowering stage[50]. The labeled spikelets were thus harvested at 2, 3, 5, 7, 10, 13, 15, 17, and 20 days after flowering (DAF). After being dehusked, samples from each of these stages comprised at least 200 seeds derived from 30 spikes that were immediately stored at -80°C until protein extraction.

### Total anthocyanin content (TAC) measurement

The reported spectrophotometric method with some modifications was used to determine the total amount of ACNs[51]. Briefly, the seeds were ground to a fine powder in liquid nitrogen. Approximately 3 g of the powder was transferred into a 50mL tube, and then a total of 24 mL of acidified ethanol (ethanol and 1.0 N HCl, 85:15, v/v) was then added for total anthocyanin content extraction. The solution was then mixed and adjusted to a pH level of 1 using 4 N HCl. The resulting solution was then shaken for 15 min, readjusted to pH 1 as required, and then again shaken for another 15 min. The tube was then centrifuged at 30,000*g* for 15 min, and the supernatant was transferred into a 50-mL volumetric flask, and acidified ethanol was added to top up to volume. Triplicate biological preparations were performed for each sample. Absorbance was measured at a wavelength of 535 nm against a blank. The TAC (in micrograms per gram) was calculated as follows:

TAC=A×28.82;

where *A* is the absorbance reading [51].

### Protein extraction and digestion

Seeds (1 g) were ground in ice-cold extraction buffer [20 mMTris-HCl (pH 8.0), 20 mM NaCl, 10 mM phenylmethylsulfonyl fluoride, and 10 mM dithiothreitol]. The supernatant was separated by centrifugation at 35,000*g*for 20 min at 4°C. The pellet was then resuspended in fresh extraction buffer for another round of extraction, and then again centrifuged at 35,000*g*for 20 min at 4°C to separate the supernatant. The proteins in the pooled supernatants were precipitated using 4 volumes of ice-cold trichloroacetic acid-acetone (10% trichloroacetic acid mixed in 100% acetone) for 4 h at -20°C,followed by isolation via centrifugation at 35,000*g* for 20 min. The protein pellet was then washed with cold 80% acetone with 0.07% (w/v) β-mercaptoethanol, followed by cold acetone with 0.07% (w/v) β-mercaptoethanol, and then vacuum-dried as previously elsewhere[52]. The homogenate was then centrifuged, and the collected pellets were air-dried, mixed in 800 μL STD buffer (4% SDS, 150 mM Tris-HCl, 100 mM DTT, pH 7.6), boiled for 5 min, and then sonicated. After centrifugation, the supernatants were then collected, and protein content was measured by using a BCA protein assay reagent (Beyotime Institute of Biotechnology, Shanghai, China). Similar amounts of proteins (120 μg from 3, 7, 10 DAF, 15, and 20 DAF) were then mixed and used as reference (REF).

Protein digestion was conducted using the FASP procedure [53]. For each sample, 300 μg of proteins were placed on an ultrafiltration filter (30 kDa cut-off, Sartorius, Gottingen, Germany) that consisted of 200 μL of UA buffer (8 M urea, 150 mM Tris-HCl, pH 8.0), centrifuged at 14,000*g* for 30 min, and then washed using 200 μL of UA buffer. Approximately 100 μL of 50 mM iodoacetamide was then added to the filter in order to block any reduced cysteine residues. The samples were then kept at room temperature for 30 min in the dark, which was followed by centrifugation at a speed of 14,000*g* for 30 min. The filters were then washed twice with UA buffer (100 μL), followed by centrifugation at 14,000*g* for 20 min after every wash. Next, approximately 100 μL of a dissolution buffer (Applied Biosystems, Foster City, CA, USA) was placed on the filter, and then centrifuged at 14,000*g* for 20 min, and then repeated twice. Then, the protein suspensions were subjected to enzyme digestion using 40 μL of trypsin (Promega, Madison, WI, USA) buffer (4 μg trypsin in 40 μL of dissolution buffer) for 16–18 h at 37°C. Last, the filter unit was then transferred to a new tube and spun at 14,000*g* for 30 min. The collected peptides were collected in the form of a filtrate, and the concentration of the peptides was analyzed at an optical density using a wavelength of 280 nm (OD_280_)[53].

### iTRAQ labeling and strong cationex change

iTRAQ labeling was conducted following the manufacturer’s recommendations (Applied Biosystems, Foster City, CA, USA). Briefly, the peptide pellet was reconstituted in 30 μL of the iTRAQ dissolution buffer. Labeling of each sample (100 μg) using the iTRAQ Reagent-8plex Multiplex Kit (AB SCIEX, Framingham, MA, USA) at 3, 7, 10, 15, and 20 DAF samples was performed twice. A REF sample was then added to each group. Then, the labeling reaction was kept at room temperature for 1 h before further analysis. From each group, 7 labeled samples were pooled and then vacuum-dried in a centrifuge at room temperature.

The iTRAQ-labeled peptides were then subjected to the procedure of strong cation exchange fractionation using an AKTA Purifier 100 (GE Healthcare, Little Chalfont, Bucks, UK), which was equipped with a polysulfethyl (PolyLC Inc., Columbia, MD, USA.) column (4.6 mm × 100 mm, 5 μm, and 200 A). The peptides were then eluted at a flow rate of 1 mL/min. Buffer A comprised 10 mM KH_2_PO_4_ and 25% v/v can (pH 3.0),whereas Buffer B contained 10 mM KH_2_PO_4_, 25% v/v ACN, and 500 mM KCl (pH 3.0). Both buffers were then filter-sterilized. The gradient utilized for separation was as follows: 100% Buffer A for 25 min, 0–10% Buffer B for 7 min, 10%–20% Buffer B for 10 min, 20%–45% Buffer B for 5 min, 45%–100% Buffer B for 5 min, 100% Buffer B for 8 min, and lastly, 100% Buffer A for 15 min. Monitoring of the elution process was performed by measuring the absorbances at a wavelength of 214 nm, with fractions collected at 1-min intervals. The collected fractions (a total of 30) were then combined into 10 pools and desalted using C18 cartridges (Sigma, Santa Clara, CA, USA). Every fraction was concentrated by vacuum centrifugation, followed by reconstitution in 40 μL of 0.1% (v/v) trifluoroacetic acid. The samples were stored at -80°C until LC-MS/MS analysis.

### LC-MSMS analysis

The iTRAQ-labeled samples were then analyzed by using the Easy-nLC nanoflow HPLC system (Thermo Fisher Scientific, Karlsruhe, BW, Germany), which was connected to an LTQ Orbitrap Elite mass spectrometer (Thermo Fisher Scientific, Karlsruhe, BW, Germany). A total of 1 μg of every sample was loaded onto an Thermo Scientific EASY column (two columns) using an autosampler, using a flow rate of 150 nL/min. Peptide sequential separation using the Thermo Scientific EASY trap column (100 μm × 2 cm, 5 μm, 100 Å, C18) and analytical column (75 μm × 25 cm, 5 μm, 100 Å, C18) was conducted using a segmented 2-h gradient of Solvent A (0.1% formic acid in water) to 35% Solvent B (0.1% formic acid in 100% ACN) for 100 min, which was then followed by 35%-90% Solvent B for 3 min, and finally, 90% Solvent B for 5 min. Then, the column was re-equilibrated to attain its initial highly aqueous solvent composition prior to analysis.

The mass spectrometer was run in a positive ion mode, wherein the MS spectra were acquired within a range of 300–2,000 m/z. The resolution of the MS and MS/MS scan using 200 m/z for the LTQ Orbitrap Elite was set to 60,000 and 15,000, respectively. The 10 signals showing the highest intensities in the acquired MS spectra were then selected for additional MS/MS analysis. The isolation window employed was 1 m/z, with ions fragmented using higher energy collisional dissociation at normalized collision energies of 35 eV. The highest ion injection times utilized were 50 ms for the survey scan and then 150 ms for the MS/MS scans, whereas the automatic gain control target values for the full scan modes were set to 1.0×10^−6^, and that for MS/MS was 5.0×10^4^. The dynamic exclusion duration was 30 s.

### Database search and protein quantification

The raw files were analyzed by using the software, Proteome Discoverer 1.4 (Thermo Fisher Scientific, Karlsruhe, BW, Germany). Identification of the fragmentation spectra was conducted using the MASCOT 2.2 search engine that was embedded in Proteome Discoverer and run against the rice protein database (released in May 2015 and including 144,386 sequences from NCBI). The search parameters were as follows: monoisotopic mass, trypsin utilized as the cleavage enzyme, two missed cleavages, iTRAQ labeling and carbamidomethylation of cysteine used as fixed modifications; and peptide charges of 2+, 3+, and 4+, as well as methionine oxidation were designated as variable modifications. The mass tolerance was 10 ppm for precursor ions and 0.05 Da for fragmented ions. The results were then filtered according to a false discovery rate (FDR) of <1%.

The relative quantitative protein analysis of samples according to the ratios of iTRAQ reporter ions derived from all unique peptides that represented each protein was conducted using the Proteome Discoverer software (version 1.4). The relative peak intensities of the iTRAQ reporter ions that were derived from each of the MS/MS spectra were employed, and the REF sample was used as a reference in calculating for the iTRAQ ratios of the reporter ions. The final ratios derived from the relative protein quantifications were then normalized according to the median protein quantification ratio. The protein ratios represented the median of the unique peptides in the protein. Only proteins identified at all two replicates were considered for further analysis.

### Bioinformatics functional analysis

Statistical and hierarchical clustering analyses were performed using Perseus V1.4.1.3 (http://141.61.102.17/perseus_doku/)[54, 55]. P-values < 0.05 by Benjamini-Hochberg FDR in Perseus and a ratio fold-change of >1.20 or <0.83 in expression between any two groups were considered significant. The hierarchical clustering analysis was performed using the following settings: Row, Column distance that was calculated using the Euclidean algorithm; Row, Column linkage–Complete. Functional analysis of the identified proteins was performed using Gene Ontology (GO) annotation (http://www.geneontology.org), and the proteins were categorized based on its biological process, molecular function, as well as cellular localization [56]. The differentially expressed proteins were then further assigned to the Clusters of Orthologous Groups of proteins (COG) database (http://www.ncbi.nlm.nih.gov/COG/) [57].

### RNA extraction and qRT-PCR analysis

Total RNA was extracted from leaves by using the TaKaRa RNAiso reagent (TaKaRa Bio, Otsu, Japan) and then treated with RNase-free DNase I (TaKaRa Bio, Otsu, Japan)[58]. The purified RNA was reverse-transcribed by using the M-MLV reverse transcription system (Promega, Madison, WI, USA), following the manufacturer’s instructions. qRT–PCR was conducted in 96-well blocks using an CFX96 Real-time System (BioRad, Hercules, CA, USA) using the SYBR Green I master mix in a volume of 25μL. Each qRT-PCR was run in triplicate. To normalize the expression data, the gene, *Actin* (GenBank Accession Number: AY212324) was used as internal reference. Relative expression levels were then calculated by using the 2^-∆∆^CT (cycle threshold) method[59].

## Results

### Physiological characterization of developing black rice grains

**Fig 1:**
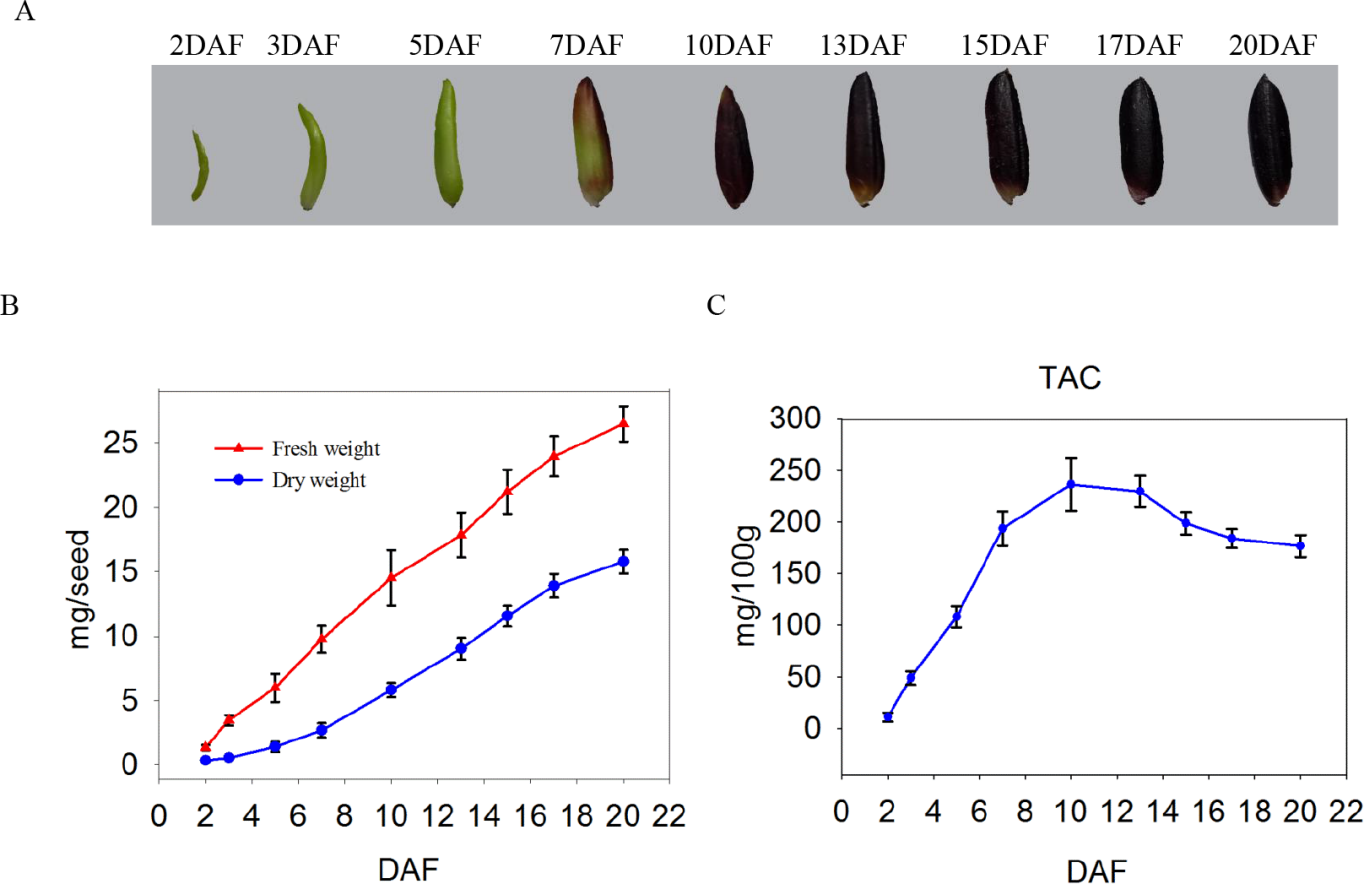
Development of black rice seed. (A) The 9 stages of seed development. (B) Changes in the fresh and dry weights of developing seeds. At least 100 seeds were examined at various stages of development. (C) Changes in total anthocyanin content (TAC) levels in developing seeds.

To generate basic information on rice seed development, we examined the morphological features as well as dynamic changes in reserve accumulation in the developing seeds at 2, 3, 5, 7, 10, 13, 15, 17, and 20 DAF (**Fig 1A and 1B**). The developing seeds showed a significant increase in size starting from 2 to 7 DAF, followed by a slight increase that apparently reached the size of mature seeds at around 13 DAF (**Fig 1A**). After 17 DAF, the seeds became black and full-filled (**Fig 1A**). On the other hand, both fresh and dry weights apparently changed insignificantly from 2 to 7 DAF, although these rapidly increased thereafter up to 17 DAF (**Fig 1B**). Beyond 17 DAF, the increase in dry weight occurred at a slower rate, although the fresh weight continued to increase until 20 DAF, thereby indicating that the developing seeds had entered the desiccation phase by 17 DAF. The developmental alterations in seed size, fresh weight, as well as dry weight were generally similar with the results of previous investigations [32, 60]. Taken together, the results of the present study indicate that developing seeds up to 7 DAF are actively dividing and differentiating, and the grains begin to fill and then ripen at 7 DAF, which then continues on up to 20 DAF. Because the goal of the present study was to conduct protein expression profiling in relation to ACN biosynthesis during grain filling, we determined the dynamic change in the TAC in seeds. The results indicated that TAC content increased from 2 to 10 DAF, which was then followed by a dramatic decrease (**Fig 1C**), thereby suggesting 2 points: (1) ACNs did not accumulate during the early stages and the grains appeared almost non-pigmented, and (2) the rates of development of the bran outer layers and endosperm differ because ACNs exist almost exclusively at the early and mid-stages, but not at the late stages. Based on these observations, we then divided the development process encompassing 2 to 20 DAF into early (2–7 DAF), mid (7–13 DAF), and late (13–20 DAF) categories, and used the developing seeds at 3, 7, 10, 15, and 20 DAF for further analysis.

### General information on iTRAQ analysis

**Fig 2:**
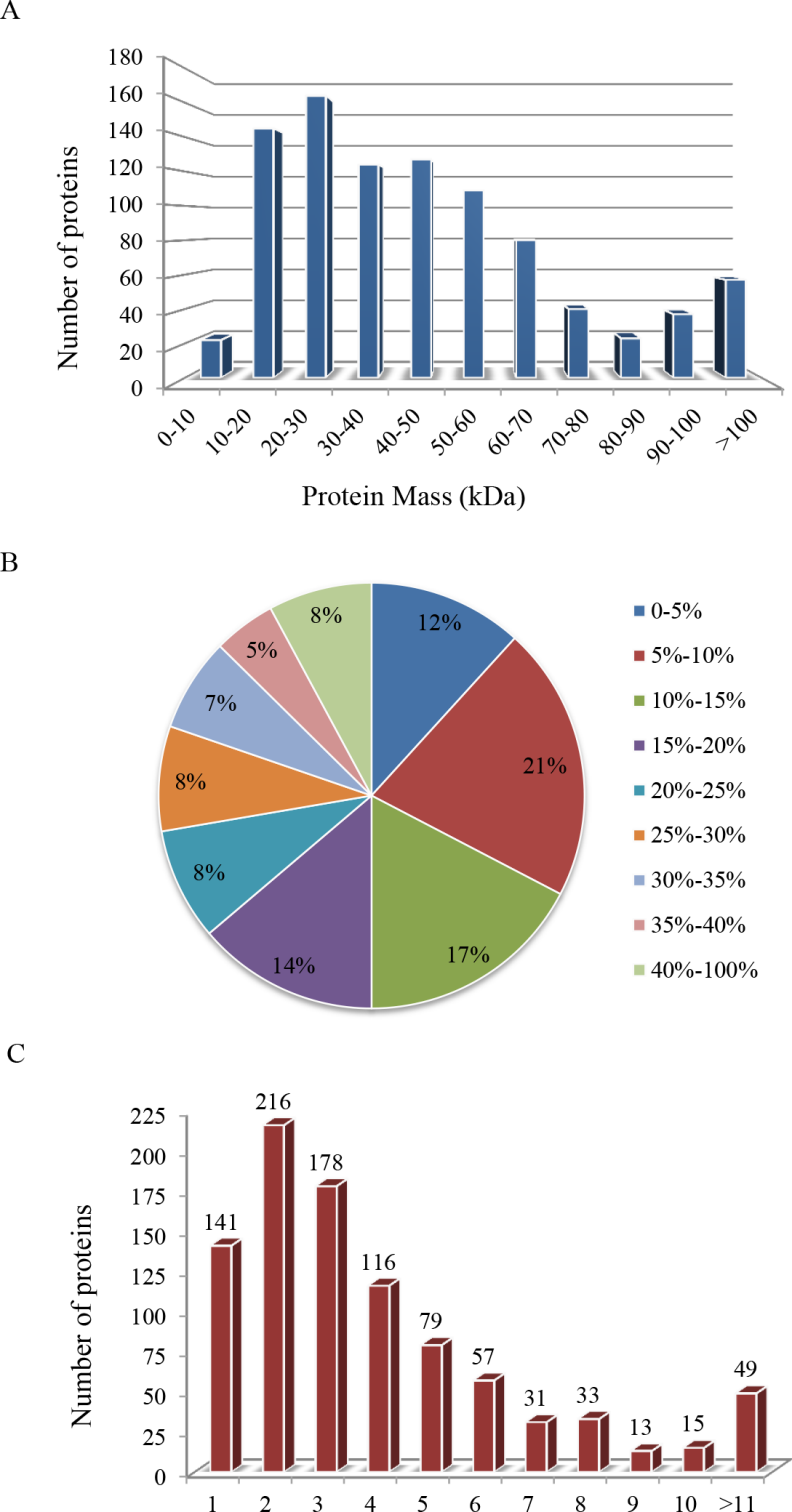
General information on the iTRAQ-LC−MS/MS analysis that was performed using black rice seed. (A) Distribution of proteins of different molecular weights. (B) Coverage of proteins by using LC-MS/MS-identified peptides. (C) Unique peptides of the detected proteins. The number of proteins in each of the categories is presented above each bar.

In the present study, a total of 2,084 proteins were identified in the experiments, which included the developmental stages and replicates of the iTRAQ proteomic analysis. Approximately 928 proteins were identified in the 2 biological replicates and were subsequently included in a comparative analysis. **Fig 2** shows the basic information that was generated from the iTRAQ-LC−MS/MS data. The molecular weights of majority of the identified proteins (80%, **Fig 2A**) were between 20 and 70 kDa. The distribution analysis of the sequence coverage of the detected peptides indicated that 88% of these proteins have a coverage that was>5% (**Fig 2B**) and that 84.81% (787) were inferred from at least 2 unique peptides (**Fig 2C**). These results thus indicate good quality data.

### Comparative analysis of protein expression at five developmental stages

**Fig 3:**
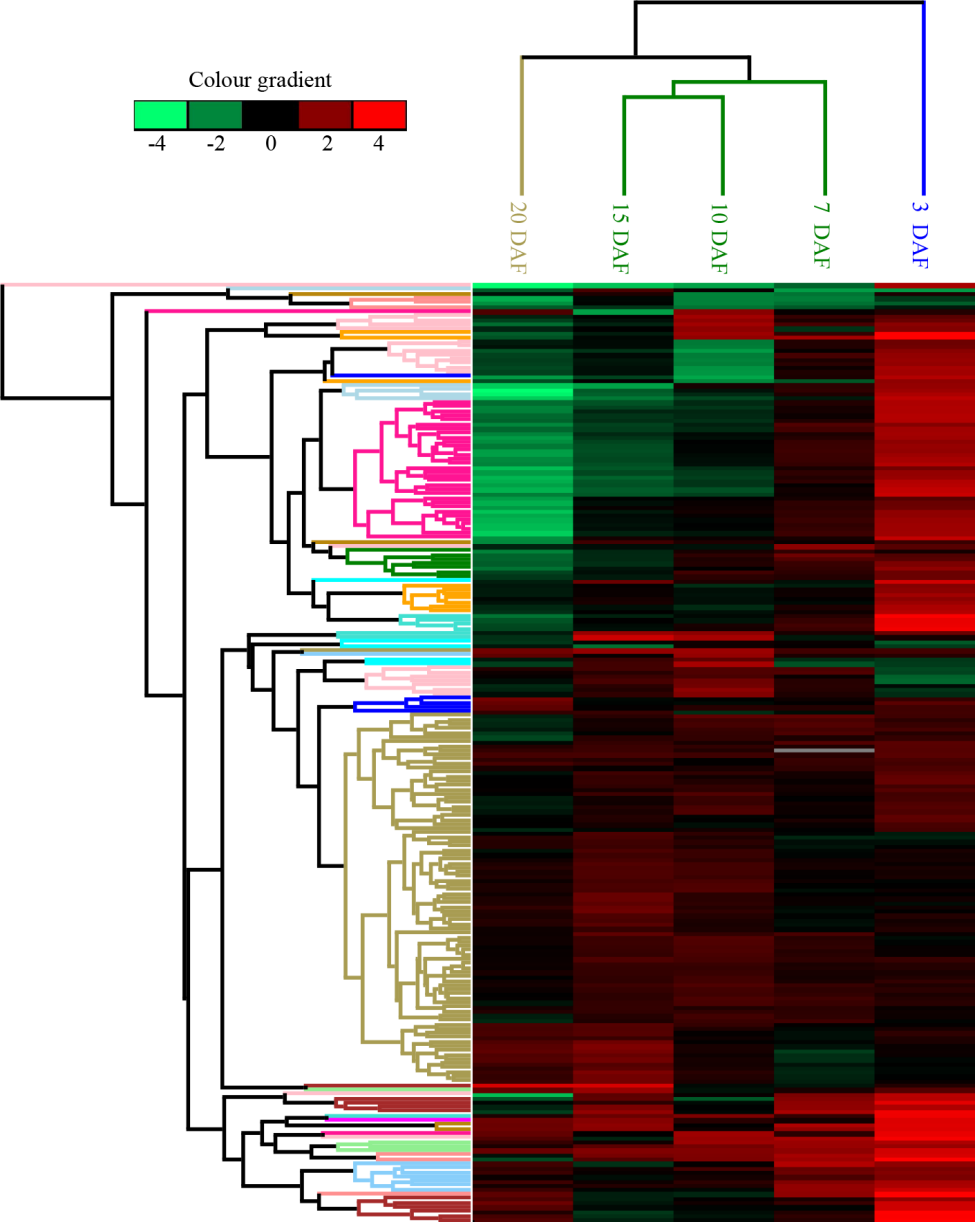
Hierarchical clustering analysis of the 230 differentially expressed proteins at 5 seed development stages. Green: Reduced expression compared to that observed in the REF. Red: Increased expression compared to that detected in the REF. White: No data.

**Fig 4:**
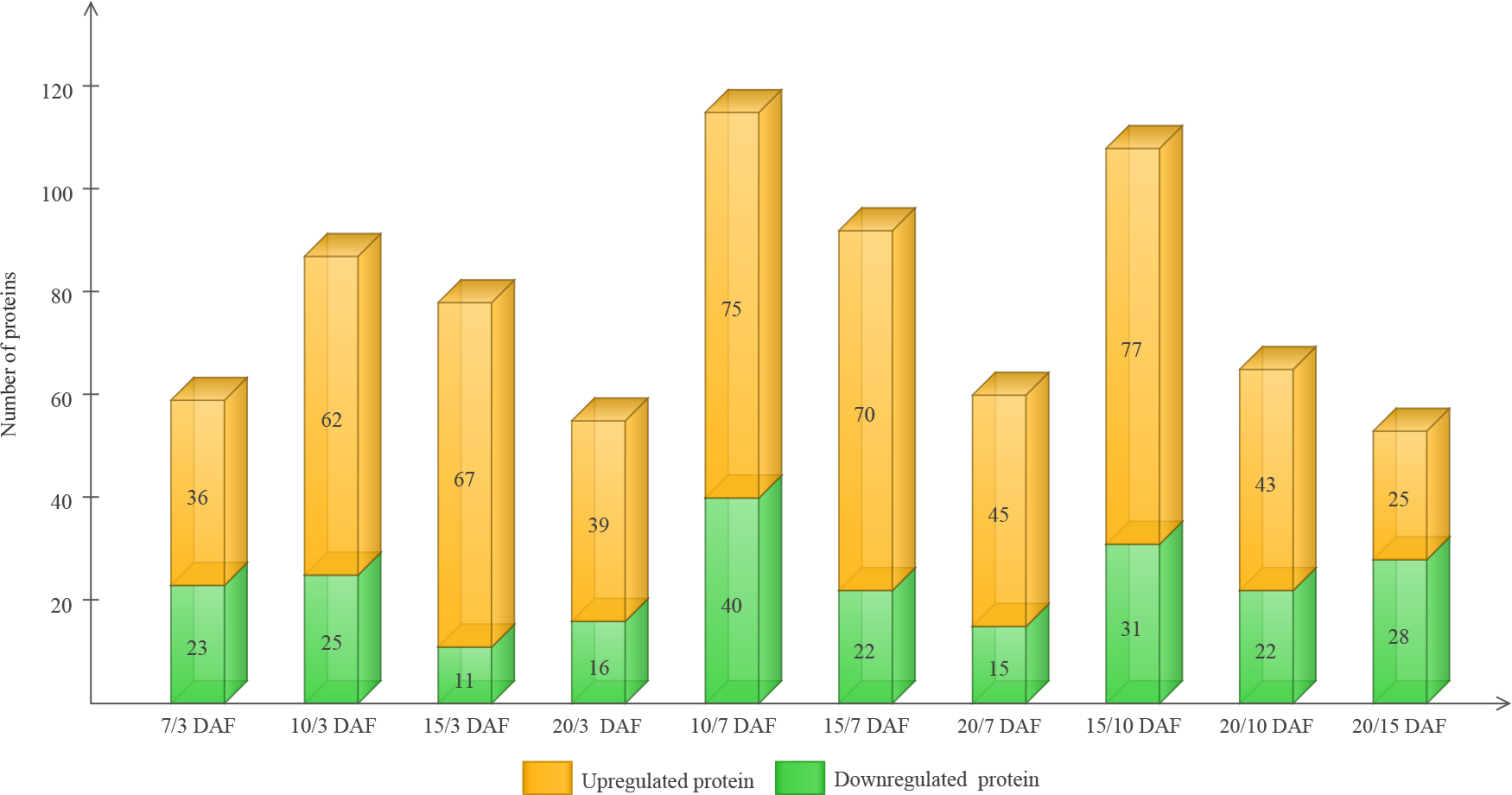
The number of differentially expressed proteins in 5 seed development stages. The x-axis indicates the comparisons between any two samples. The left y-axis shows the number of differentially expressed proteins.

Using a cutoff of P-value <0.05 and a ratio fold-change of>1.20 or <0.83 for expression, we identified 230 differentially expressed proteins after comparing at least 1 sample pair (**S1 Table**). These 230 proteins were grouped based on fold-change in expression, as visualized in **Fig 3**. The analysis revealed 2 main branches. DAF 3 was distinct from DAF 7, 10, 15, and 20. Similarly, DAF 7, 10, 15, and DAF 20 were also distinguishable from each other, and could be resolved into 2 separate clusters within the same branch. The maximum number of differentially expressed proteins was observed in the sample pair consisting of the time points of 7 and 10 DAF (76 upregulated and 39 downregulated proteins), followed by the sample pair made up of the time points of 10 and 15 DAF (77 unregulated and 28 downregulated proteins) (**Fig 4**). This finding indicates that a distinct metabolic phase could possibly serve as an indicator of developmental reprogramming. In contrast, the sample pair consisting of the time points of 15 and 20 DAF presented the lowest number of differentially expressed proteins (25 upregulated and 28 downregulated proteins) (**Fig 4**), which may be attributable to the low level of metabolic activity in cells during grain development.

#### Classification of metabolic process-specific proteins

**Fig 5:**
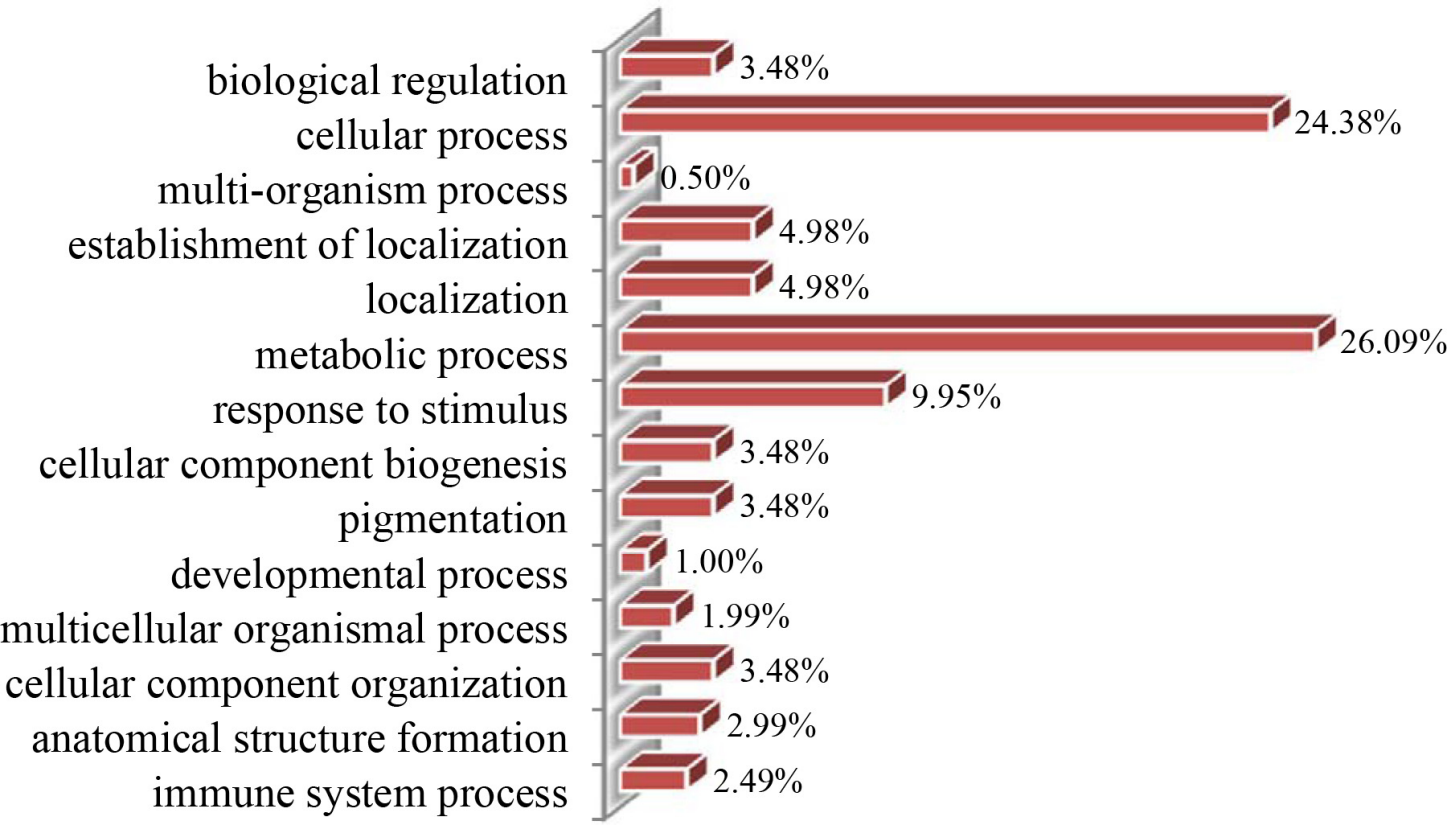
Biological process classification of differentially accumulated proteins during grain development.

**Fig 6:**
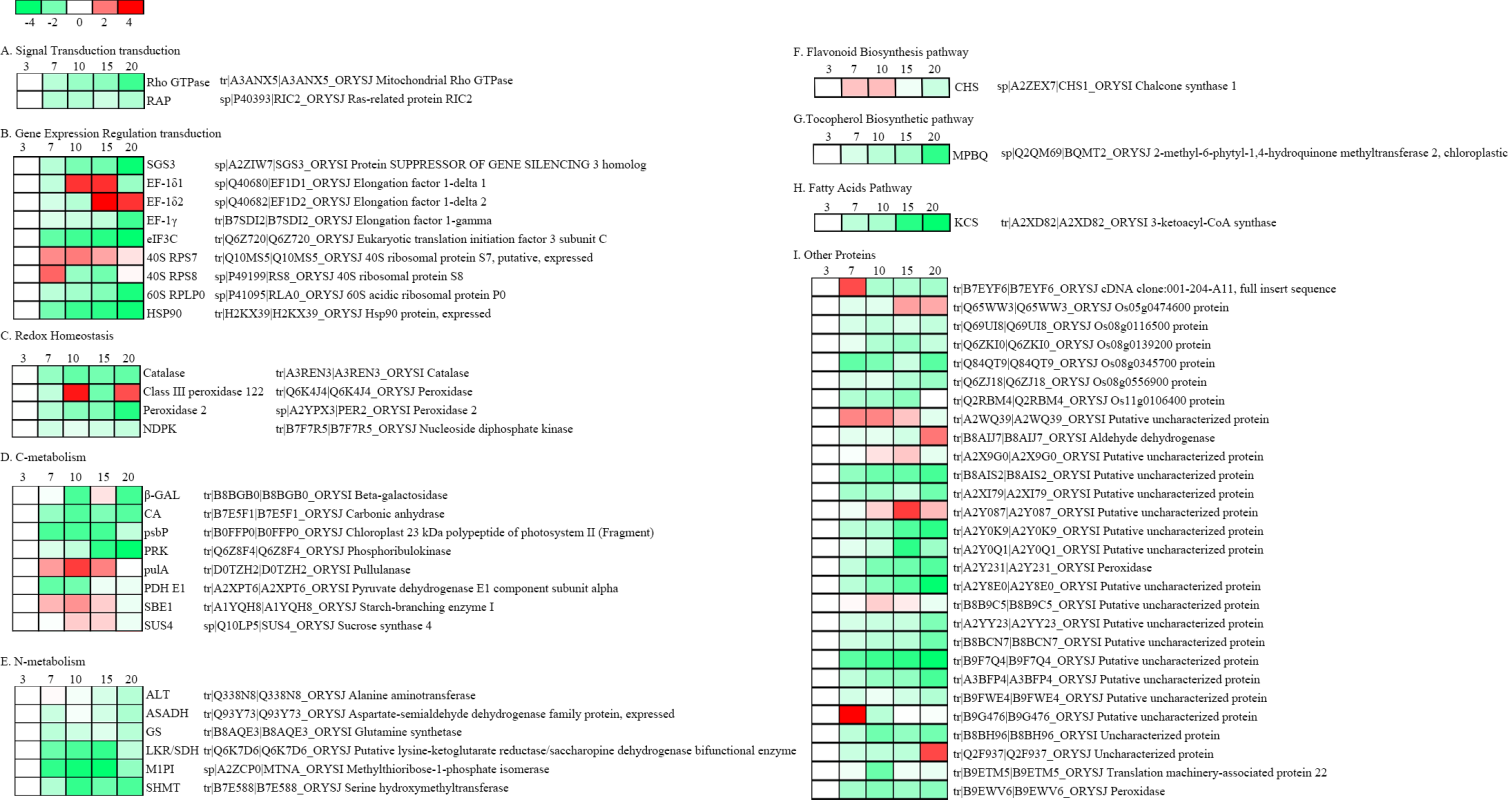
Profiles of the functional protein clusters in relation to black rice grain development. A heat map representing the log_2_ relative abundance of differentially expressed proteins during grain development in relation to the 3 days after flowering (DAF) stages was generated using Perseus (v1.4.1.3) and using the iTRAQ-derived quantitative data. The abbreviation for each protein is provided, using the gene index accession number as well as the sequence description assigned using UniProt. Proteins were grouped according to their known or putative role in metabolic pathways or cellular processes.

The 230 differentially accumulated proteins were categorized into 3 groups (biological process, cellular component, and molecular function) based on the results of GO analysis. According to the biological process properties (**Fig 5**), the main functional categories were metabolic process (26.09%), cellular process (24.38%), response to stimulus (9.95%), localization (4.98%), establishment of localization (4.98%), cellular component organization (3.48%), pigmentation (3.48%), cellular component biogenesis (3.48%), biological regulation (3.48%), anatomical structure formation (2.99%), immune system process (2.49%), multicellular organismal process (1.99%), developmental process (1.0%), and multi-organism process (0.5%). The most represented GO term was ‘metabolic processes’, with 60 proteins differentially regulated during black rice grain development. Through KEGG (http://www.kegg.jp/), Metacyc (http://metacyc.org/), and UniPathway (http://www.grenoble.prabi.fr/obiwarehouse/unipathway) analyses, as well as a review of literature indicated that the 60 differentially expressed proteins of the metabolic process GO category were assigned to 9 subgroups (**Fig 6**).

### Validation using quantitative RT-PCR

**Fig 7:**
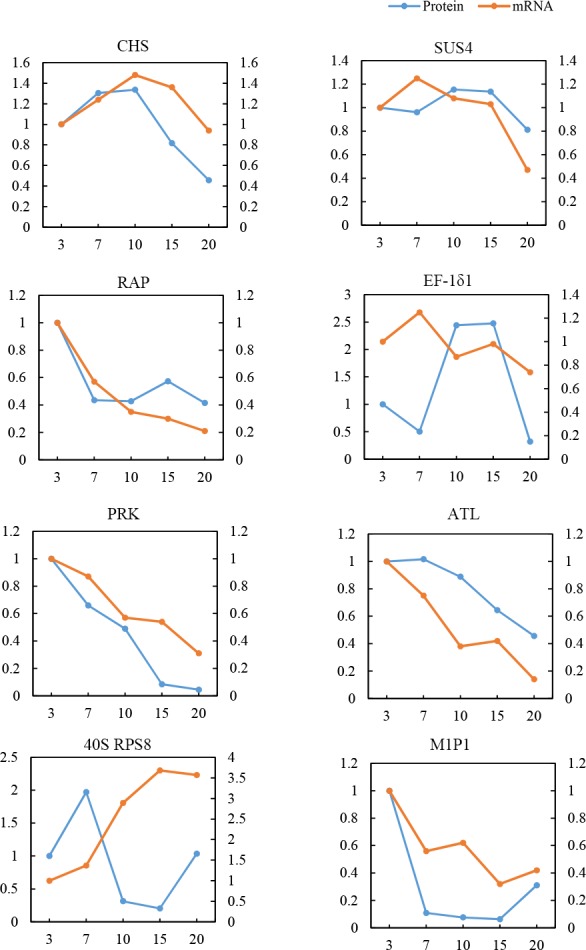
Comparative analysis of protein and mRNA profiles of 8 representative metabolic proteins. The x-axis represents days after flowering (DAF). The left y-axis indicates the relative protein level, whereas the right y-axis pertains to the relative mRNA level. The blue line represents the pattern of protein expression, and the orange line indicates the pattern of mRNA expression.

To validate the quantitative results relating to the correspondence between proteins and its mRNA expression patterns, 8 metabolic process proteins were performed to evaluate the dynamic transcriptional expression profiles by using quantitative RT-PCR (Q-PCR) analysis. The relatively high amplification efficiency of every primer pair was designed using the software, primer-BLAST[61], and the sequences are presented in Table 1. **Fig 7** shows that 2 genes encoding chalcone synthase 1 (CHS) and methylthioribose-1-phosphate isomerase (M1P1) displayed similar protein expression patterns, and four genes encoding sucrose synthase 4 (SUS4), Ras-related protein RIC2(RAP), phosphoribulokinase (PRK), and phosphoribulokinase (ALT) showed expression patterns at 5 developmental stages that were similar to its protein expression profiles. On the other hand, 2 genes encoding elongation factor 1-delta 2 (EF-1δ1) and 40S ribosomal protein S8 (40S RPS8) showed expression patterns that were opposite to that of its proteins. The mRNA land protein levels of EF-1δ1 and 40S RPS8 differed and may be attributable to translational or post-translational modifications.

**Table 1:** Design of primers used for quantitative PCR analysis.

Uniprot ID	Genesymbol	Forward primer (5′-3′)	Reverse primer (5′-3′)
A2ZEX7	*CHS*	GGAGGCGAAGGTTGGGCT	GTGATGGGGACGCTGTGG
Q10LP5	*SUS4*	AGGCACACTGACTGTAGGTCCT	AGTTACATTCCATTTTATTATCGG
P40393	*RAP*	GCGTTGCTTGTCTATGATGTC	AACTATGTTTGGGTCTGTATGGT
Q40680	*EF-1δ1*	GTCCTCAGTGTTGCTTGATGTC	TGGTTCGGTGTAGAAGTAGTCC
Q6Z8F4	*PRK*	AGGACTGGCAGGAAGGAGAAAG	TGGGATGTAAGCCCTCAATGAC
Q338N8	*ATL*	ATTGATGTTAGGGCTTTGGTAGTT	AGTTCACTGACGCCACTTTGTAGA
P49199	*40S RPS8*	AGCGAAGAAAAGCAACCAT	ACCTCCCACTGCCAAACTG
A2ZCP0	*M1PI*	CTCCAGTCCATCGTCTACCACC	ACCCAGAAACCTCTACAGCCAG

## Discussion

The pericarp of black rice seeds consists of high levels of ACN. However, information regarding metabolic mechanisms underlying ACN biosynthesis is limited. In the present study, iTRAQ-based quantitative proteome analysis indicated the occurrence of dynamic changes in metabolic process proteins of black rice seeds at 5 developmental stages. The number of identified metabolic proteins was significantly higher than that observed in previous reports using traditional gel-based approaches [36, 62, 63]. These metabolic proteins were mainly involved in C-metabolism, N-metabolism, as well as the flavonoid biosynthesis pathway.

### Proteins involved in signal transduction

**Fig 8:**
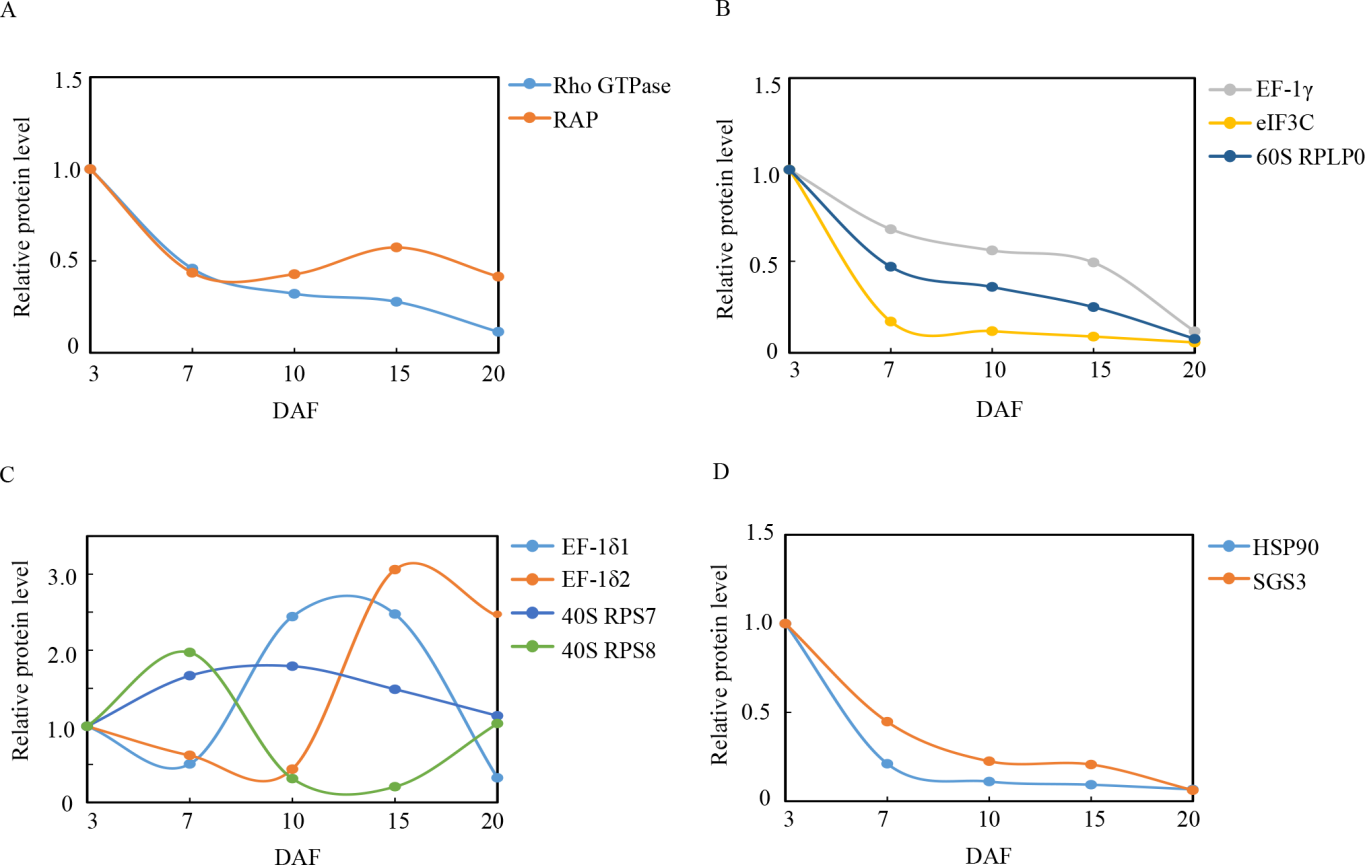
Relative expression patterns of signal transduction, as well as gene expression regulation proteins during grain development. The expression level was established in relation to 3 days after flowering (DAF) (vertical axis) at various developmental stages (horizontal axis, DAF).

Black rice grains perceive ACN biosynthesis signals by using putative sensors, which are then transmitted to the cellular machinery via signal transduction in order to regulate gene expression. Two proteins involved in signal transduction were detected and quantified in the present study, namely, mitochondrial Rho GTPase (Rho GTPase) and Ras-related protein RIC2 (RAP). These findings suggest that the Rho family GTPases, as well as RAP act as key molecular switches that control a variety of actin-based cellular processes, including the establishment of cell polarity, cellular morphogenesis, and motility of diverse eukaryotic organisms [64]. Rho GTPase and RAP were upregulated at 3 DAF and were downregulated during rice grain ripening (**Fig 8A**). The observed changes in the characteristics of these 2 proteins and the TAC trend were not entirely the same, which may be due to the fact that the signal transduction pathway is relatively complex. The identification of novel components expands our knowledge regarding ACN biosynthesis during black rice grain development.

#### Proteins involved in gene expression regulation

ACN biosynthesis undergoes fine temporal and spatial regulation involving numerous levels of regulation, including transcriptional or translational control [65]. Regulation of gene expression through translational control is common in various organisms. In terms of protein synthesis processes, mRNA translation is controlled at both global as well as message-specific levels, particularly during translation initiation [66]. Elongation factors 1-delta 1 (EF-1δ1), -delta 2 (EF-1δ2,), 1-gamma (EF-1γ), eukaryotic translation initiation factor 3 subunit C (eIF3C), 40S ribosomal protein S7(40S RPS7) and S8(40S RPS8), and 60S acidic ribosomal protein P0 (60S RPLP0) were all involved in the translation and processing of proteins. These findings suggest that eukaryotic elongation factors (eEFs) are involved in the translation elongation cycle, which deliver aminoacyl-tRNAs (aa-tRNAs) to the elongation ribosome. Although EF-1γ, eIF3C, and 60S RPLP0 were downregulated (**Fig 8B**), EF-1δ1, EF-1δ2, 40S RPS7, and 40S RPS8 were upregulated during DAF10,15,10, and7, respectively (**Fig 8C**). The differential regulation of various components of the translation machinery is indicative of a complicated mechanism of controlling protein synthesis during grain development. Heat shock proteins function in minimizing the aggregation of newly synthesized proteins, thereby facilitating the folding process s[67]. Heat shock protein 90 (HSP90) was downregulated during grain development (**Fig 8D**), which in turn may play a critical role in preventing clumping of denatured proteins and in facilitating protein refolding during grain development. In plants, post-transcriptional gene silencing (PTGS) involves the reduction in the levels of specific RNAs upon the introduction of homologous sequences into the plant genome. This particular reduction is due to an increase in turnover of target RNA species, while transcription levels of corresponding genes are unaltered [68]. In the present study, a protein suppressor of gene silencing 3 (SGS3) was identified and quantified, which was downregulated during rice grain development (**Fig 8D**). However, the role of SGS3 in rice grain development remains elusive.

### Proteins involved in redox homeostasis

**Fig 9:**
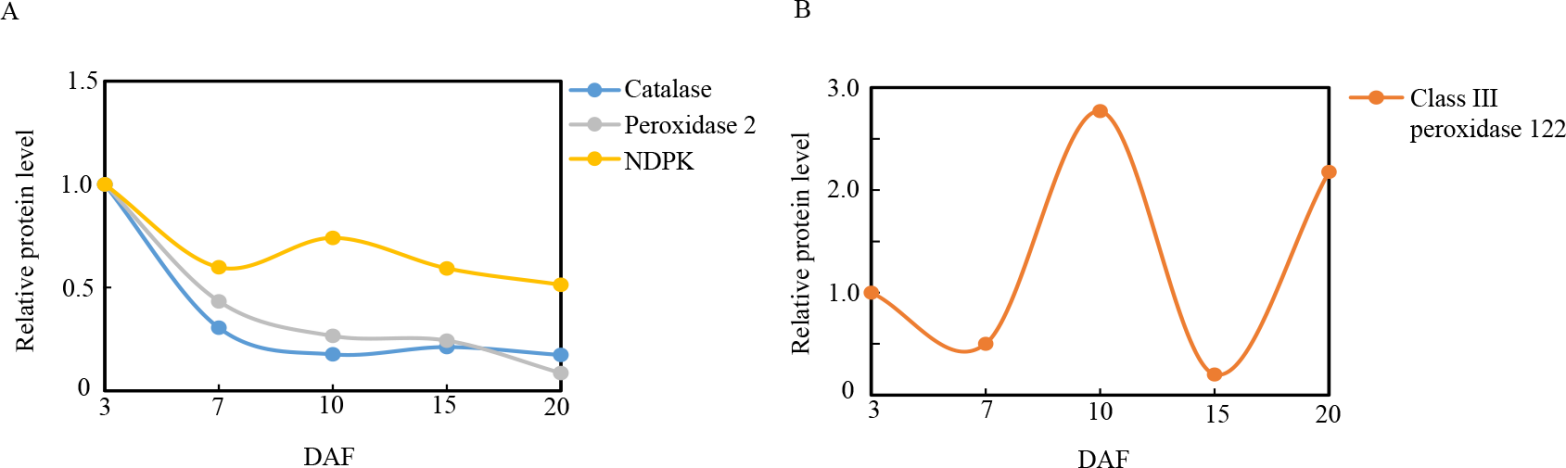
Relative expression patterns of redox homeostasis proteins during grain development. The expression level was established in relation to 3 days after flowering (DAF) (vertical axis) at various developmental stages (horizontal axis, DAF).

Redox homeostasis pertains to the metabolic interface that links stress perception with physiological responses [69]. Previous studies have shown that the early signaling events that control transcription factor expression, which in turn coordinates and regulates the entire flavonoid biosynthesis pathway, are redox-sensitive [70]. Plants regulate redox homeostasis using sophisticated mechanisms, including scavenging radicals by using peroxidases, (i.e., catalase, peroxidase 2, and class III peroxidase 122) and a nucleoside diphosphate kinase (NDPK), which were all identified in the present study. Nucleoside diphosphate kinase (NDPK) is a multifunctional protein that is involved in cell proliferation, development, as well as differentiation in eukaryotes. Aside from its basic enzymatic role involving phosphor-transfer as well as regeneration of nucleoside triphosphates, NDP kinase 2 is a component of the H_2_O_2_-activated MAPK signaling pathway in plants and its upregulation alters cellular redox conditions. The expression of NDPK2 is induced by H_2_O_2_, and knockout of NDPK2 results in increased ROS accumulation and stress sensitivity. On the other hand, upregulation of NDPK2 results in a decrease in H_2_O_2_levels and sensitivity to stress [71]. Catalase, peroxidase 2, and NDPK were downregulated during ripening (**Fig 9A**), whereas class III peroxidase 122 was upregulated by 2.76-fold in DAF10, sharply decreased in DAF15, and then upregulated by 2.18-fold at DAF20 (**Fig 9B**). The increase in class III peroxidase 122 activity directly correlated with protein abundance in proanthocyanidin-deficient *A*. *thaliana* seeds[72], and the increase in class III peroxidase 122 in DAF10 is correlated with changes in ACN levels. The pattern of antioxidant enzyme expression in grain development demonstrates that when the levels of catalase and peroxidase 2 activity decrease, class III peroxidase 122 (PODs) activity takes over to maintain redox homeostasis.

### Proteins involved in C-metabolism

Carbohydrates, or sugars, play a major role in plant growth. Recent studies have shown the stimulatory effects of sugars on the synthesis of ACNs in various organs of numerous plant species [73]. Previous investigations have proven that ACN biosynthesis occurs in plants that are germinated or grown on a sugar-containing medium [74–76]. In particular, the *CHS* gene of petunia (*Petunia hybrida*) petals in transgenic *Arabidopsis* leaves is activated by sugars [77], and petunia corollas that are cultured *in vitro* in the absence of sucrose (Suc) do not develop any pigmentation [78]. Tsukaya and Ohtoreported that *Arabidopsis* cultured on a Suc-containing medium produced high amounts of ACNs [77, 79]. Therefore, sugar content may play an essential role in ACN accumulation. In the present study, proteins involved in sugar metabolism included beta-galactosidase (β-GAL), sucrose synthase 4 (SUS4), pullulanase (pulA), and starch-branching enzyme I (SBE I).

**Fig 10:**
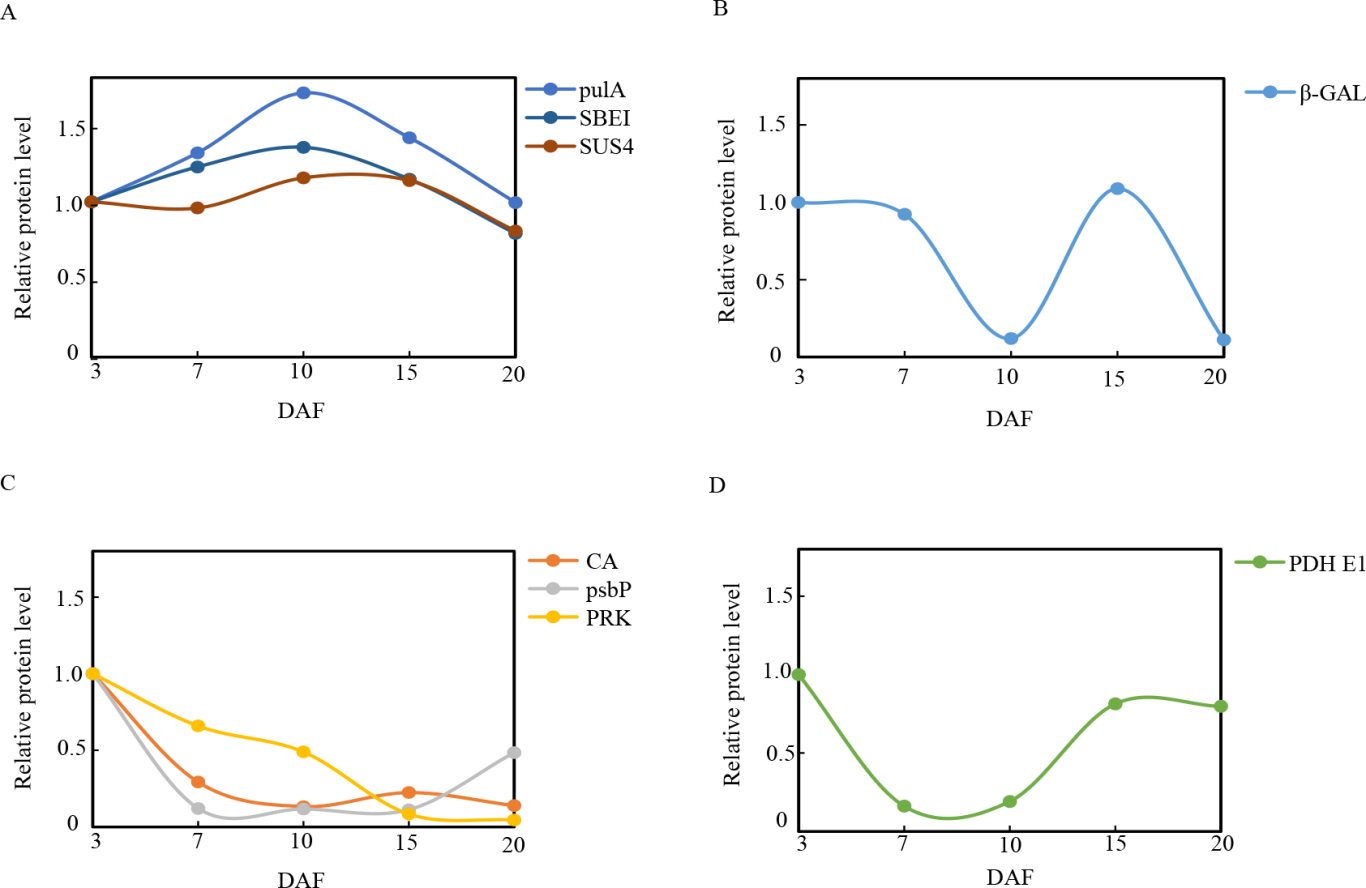
Relative expression patterns of C-metabolism proteins during grain development. The expression level was established in relation to 3 days after flowering (DAF) (vertical axis) at various developmental stages (horizontal axis, DAF).

Grain development pertains to the synthesis and accumulation of starch in endosperm cells. Sucrose is continuously cleaved into UDP-glucose and utilized in starch synthesis. Starch biosynthesis is initiated using a substrate of ADP-glucose (ADP-Glu), which is formed by AGPase. ADP-Glu is transported into the cytoplasm via an ADP-glucose brittle-1 transporter. Various classes of sucrose synthase (SS) are recruited in order to lengthen the glucan chain, including SS 1–4 and granule-bound starch synthase, whereas starch-branching enzymes (SBEs) are involved in the formation of alpha-1,6-glucoside, and starch-debranching enzymes (DBEs) hydrolyze α-(1,6) linkages of a polyglucan[80]. Three enzymes associated with sugar synthesis were detected in the present study, including SS4, SBE1, and Pullulanase[81]. All of these enzymes achieved the highest levels of expression at 10 DAF (**Fig 10A**). This is in agreement with the observed changes in ACN content. The β-GAL enzyme is an O-glycosyl hydrolase that hydrolyzes the glycosidic bond between 2 or more carbohydrates or between a carbohydrate and a non-carbohydrate moiety [82]. The present study demonstrated that β-GAL has a higher expression level at 3 DAF, but is dramatically downregulated at 10 DAF (**Fig 10B**), which could contribute to glucose accumulation and improve sucrose content during grain development. This result is concordant with previous reports that indicate that the onset of sucrose accumulation was accompanied by a decrease in β-GAL activity [83].

During the early grain stage, photosynthesis generates the raw material, triosephosphate, for starch biosynthesis, which plays an essential role in the maintenance of endogenous O_2_ balance [84]. The results of the present study showed that carbonic anhydrase (CA), chloroplast 23-kDa polypeptide of photosystem II (psbP), and phosphoribulokinase(PRK), which are necessary for photosynthesis, were upregulated at 3 DAF, particularly during the early grain development stages (**Fig 10C**), which in turn promoted plant photosynthesis as well as grain filling. The decline in the expression of these 3 enzymes during the later state of filling was suggestive of a down regulation of photosynthesis (**Fig 10C**), which has also been reported in tomatoes [85], apricots [86], grapes [87, 88], and palm [89]. The down regulation of proteins related to photosynthesis is correlated to the reduction in pyruvate dehydrogenaseE1 component (PDH E1). The PDH E1 component is a major enzyme that catalyzes oxidative decarboxylation of pyruvate to acetyl CoA, which serves as the entry point of carbohydrates directly into the Krebs cycle [90]. In the present study, PDH E1 was downregulated on DAF7 and10 (**Fig 10D**). This may indicate that while the photosynthetic rate declined, the decrease in abundance of the PDH E1 component reduced sugar consumption, which might be a signal that induces the acceleration of ACN biosynthesis [73].

### Proteins involved in N-metabolism

Plant growth and development are controlled by the concerted activities of signaling pathways, which are triggered by different environmental conditions as well as developmental cues. Nutrient availability, particularly N, is one of the major factors that regulate plant metabolism and development [91]. N limitation induces ACN accumulation in plants, which in turn influences phenylpropanoid metabolic flux as well as switches ACN biosynthesis to lignin biosynthesis [92]. Proteins involved in N-metabolism that were identified in our study included glutamine synthetase (GS), alanine aminotransferase (ATL), aspartate-semialdehyde dehydrogenase (ASADH), lysine-ketoglutarate reductase/saccharopine dehydrogenase bifunctional enzyme (LKR/SDH), methylthioribose-1-phosphate isomerase (M1PI), and serine hydroxymethyltransferase (SHMT).

**Fig 11:**
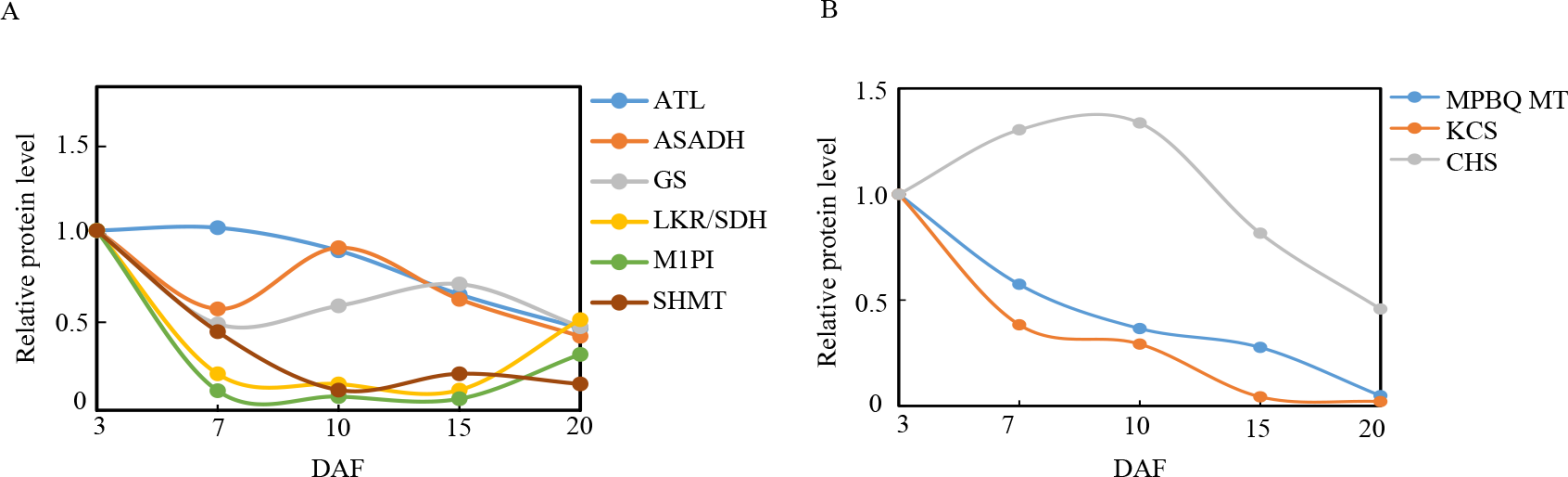
Relative expression patterns of N-metabolism and functional proteins during grain development. The expression level was established in relation to 3 days after flowering (DAF) (vertical axis) at various developmental stages (horizontal axis, DAF).

GS is triggers the first step of ammonium assimilation and transformation this into glutamine and glutamate, which are the major compounds of the amino acid biosynthetic pathway [93]. More than 95% of inorganic N in higher plants is assimilated and converted to glutamate and glutamine through the glutamine synthetize pathway, and then utilized in the generation of other amino acids during the catalysis of aspartate aminotransferase and alanine aminotransferase (ATL)[94]. The present study demonstrated that GS and ATL had a lower expression level at DAF 7 and 10 (**Fig 11A**), which was consistent with the findings of a proteomics study conducted by Zhang [95]. The decrease in GS and ATL during grain development may be attributable to N insufficiency and upregulated ACN synthesis and accumulation.

In plants, ASADH produces the branch point intermediate that occurs between the lysine and threonine/methionine pathways [96]. LKR/SDH is a bifunctional enzyme that catalyzes the first two steps of plant lysine catabolism, which controls the accumulation of different catabolic products of Lys[97]. M1PI functions in methionine salvage pathways in plants [98]. SHMT is a tetramer that induces the reversible conversion of serine and tetrahydrofolate (THF) into Gly and 5, 10-methylene THF [99]. The expression of 4 proteins (ASADH, LKR/SDH, M1PI, and SHMT)decreased during grain development(**Fig 11A**), which may contribute to insufficiency in N and induce ACN accumulation in black rice grains.

### Proteins involved in the flavonoid biosynthesis pathway

ACNs are generated by the phenylpropanoid pathway, which is initiated by the conversion of phenylalanine into cinnamic acid via phenylalanine ammonia lyase (PAL), and then diverges into various branches at ρ-coumaroyl CoA. One branch involves the flavonoid pathway, where in chalcone synthase (CHS) catalyzes the formation of the flavonoid skeleton, which is derived from ρ-coumaroyl CoA, and in turn leads to the synthesis of flavonols, cyanidins, and ACNs[92]. The present study showed that CHS was upregulated, peaking at DAF10, which corresponded to the middle of the grain development process. This protein was then downregulated at DAF20, when the development process was completed (**Fig 11B**). This result was correlated with the observed expression level of CHS in *P*. *dactylifrea*[89], and the changing trends of ACN contents in the present study.

### Proteins involved in the tocopherol biosynthetic pathway

Tocotrienols pertain to the primary form of vitamin E present in seeds of majority of monocot plants, including cereals (e.g., rice and wheat) [100]. 2-Methyl-6-phytyl-1,4-hydroquinone methyltransferase 2 (MPBQ MT) is a major component enzyme that is involved in the key methylation step in tocopherol (vitamin E) synthesis, which mediates the conversion of 2-methyl-6-phytyl-1,4-hydroquinone into 2,3-dimethyl-6-phytyl-1,4-hydroquinone and 2-methyl-6-solanyl-1,4-benzoquinoneto plastoquinone. Recent studies on the tocopherol-deficient *vte1* mutants of *A*. *thaliana* indicated that the down regulation of tocopherol increases jasmonic acid levels in plants, whereas ACN biosynthesis in epidermal, vascular, and mesophyll cells are upregulated by jasmonic acid [101, 102]. In the present study, MPBQ MT was increasingly downregulated from 3 to 20DAF (**Fig 11B**), which may have induced ACN biosynthesis by upregulating jasmonic acid levels.

### Proteins involved in fatty acid pathways

Very long-chain fatty acids (VLCFAs) are essential precursors of cuticular waxes as well as aliphatic suberins in plants. The condensation of C units into an acyl CoA via 3-ketoacyl CoA synthase (KCS) is the first committed step of VLCFA biosynthesis[103]. In the present study, KCS was downregulated during rice grain development (**Fig 11B**). The observed decrease in KCS was correlated with a reduction in fatty acid synthesis during grain development.

## Conclusions

**Fig 12:**
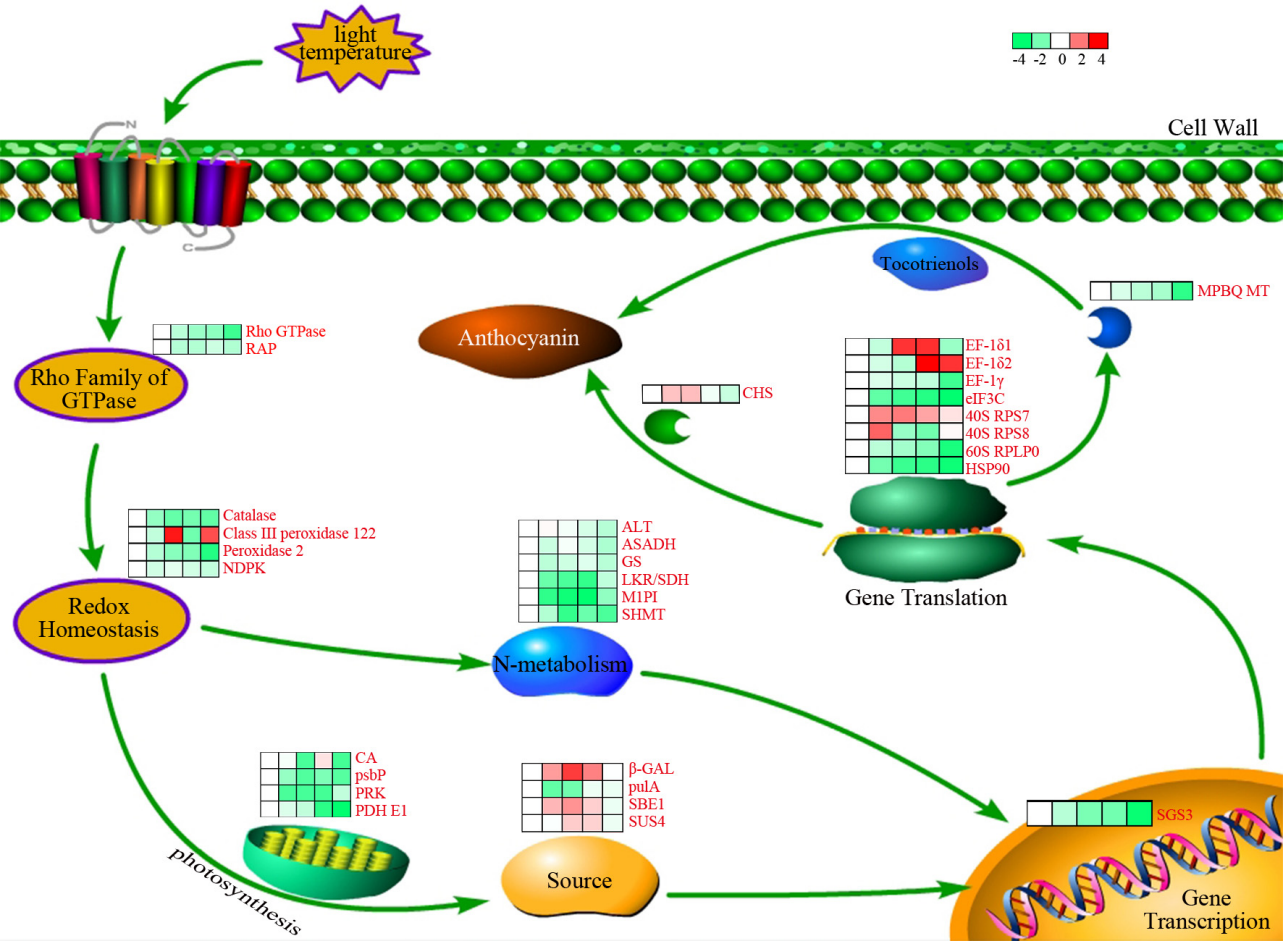
Quantification of black rice grain proteins that are related to ACN biosynthesis. RAP, Ras-related protein RIC2; NDPK, Nucleoside diphosphate kinase; CA, Carbonic anhydrase; psbP, Chloroplast 23-kDa polypeptide of photosystem II; PRK, Phosphoribulokinase; PDH E1, Pyruvate dehydrogenase E1 component subunit alpha; β-GAL, Beta-galactosidase; pulA, Pullulanase; SBE1, Starch-branching enzyme I; SUS4, Sucrose synthase 4; ALT, Alanine aminotransferase; ASADH, Aspartate-semialdehyde dehydrogenase family protein; GS, Glutamine synthetase; LKR/SDH, Putative lysine-ketoglutarate reductase/saccharopine dehydrogenase bifunctional enzyme; M1PI, Methylthioribose-1-phosphate isomerase; SHMT, Serine hydroxymethyltransferase; SGS3, Protein SUPPRESSOR OF GENE SILENCING 3 homolog; MPBQ MT, 2-methyl-6-phytyl-1,4-hydroquinone methyltransferase 2; EF-1δ1, Elongation factor 1-delta 1; EF-1δ2, Elongation factor 1-delta 2; EF-1γ, Elongation factor 1-gamma; eIF3C, Eukaryotic translation initiation factor 3 subunit C; 40S RPS7, 40S ribosomal protein S7; 40S RPS8, 40S ribosomal protein S8; 60S RPLP0, 60S acidic ribosomal protein P0; HSP90, Hsp90 protein; CHS, Chalcone synthase 1. The protein levels of deregulated enzymes are indicated by colored squares that represent changes in expression (log_2_ ratio) at various developmental stages in relation to the 3 DAF stage. In a sequence order (left to right), stages are presented from 3, 7, 10, 15, and 20 DAF.

In summary, we investigated the dynamic changes in protein expression levels during five sequential developmental stages that were associated with black rice grain filling, namely from 3 to 20 DAF. The results of the present study indicated that during plant development, proteins involved in both flavonoid and starch synthesis are upregulated, and proteins that were related to other categories and subcategories were downregulated. Importantly, the present study revealed that CHS is a key enzyme in the ACN biosynthetic pathway in black rice. In addition, we determined that a high sugar content and deficiency in N metabolism promoted ACN biosynthesis. Our results also showed that the coordination of various metabolic and cellular processes is related to ACN synthesis and accumulation during seed development. A simple model of pathways associated with ACN biosynthesis is presented in Fig 12. The results of the present study provide novel clues that facilitate in better understanding the metabolic network that is involved in ACN accumulation in developing seeds.

## Supporting Information

S1 Table Differentially expressed proteins in black rice grains at five developmental stages. (XLSX)
